# Impact of Chemical Post-Processing in Fused Deposition Modelling (FDM) on Polylactic Acid (PLA) Surface Quality and Structure

**DOI:** 10.3390/polym11030566

**Published:** 2019-03-26

**Authors:** Ana P. Valerga, Moises Batista, Severo R. Fernandez-Vidal, Antonio J. Gamez

**Affiliations:** Department of Mechanical Engineering and Industrial Design, School of Engineering, University of Cadiz, Av. Universidad de Cadiz, 10, E-11519 Puerto Real, Cadiz, Spain; moises.batista@uca.es (M.B.); raul.fernandez@uca.es (S.R.F.-V.); antoniojuan.gamez@uca.es (A.J.G.)

**Keywords:** additive manufacturing, organic solvent, chemical treatment, roughness, finishing processes, biodegradable polymer, green technology

## Abstract

The application of techniques to improve the surface finish of pieces obtained by fused deposition modelling, as well as other functional aspects, is of great interest nowadays. Polylactic acid, a biodegradable material, has been considered a possible substitute for petroleum-based polymers. In this work, different chemical post-processing methods are applied to polylactic acid pieces obtained by fused deposition modelling and some characteristics are studied. Structural, thermal, and crystallinity property changes are analyzed according to the treatments applied. This can prevent degradation, eliminate the glass transition phase of the material, and thereby increase the thermal resistance by about 50 °C. An improvement in the roughness of the pieces of up to 97% was also found.

## 1. Introduction

Polymeric additive manufacturing is one of the main topics of the strategic discussions about Industry 4.0 of the European Factories of the Future Research Association (EFFRA), due to its capacity for personalization of parts, its high flexibility, and its efficiency in the use of resources. However, the European Union has made the commitment to make all plastic packaging reusable or recyclable in a cost-effective manner by 2030 [1]. For this reason, the study of biodegradable materials for manufacturing processes is of utmost importance.

Specifically, polylactic acid (PLA) is the most widely used biodegradable polymer in the fused deposition modelling (FDM) process. In addition, this material has played a central role in the replacement of fossil-based polymers for certain applications as it is a fully aliphatic polymer. Although there are few studies on the behavior of this polymer in this technology, PLA is similar to other petroleum-based materials (e.g., *acrylonitrile butadiene styrene*) and could be substituted in many applications [2,3], as it has similar mechanical properties, such as ultimate tensile strength, hardness, and processability [4,5].

One of the main restrictions of FDM is the poor surface quality or characteristic texture [6,7]. Since the inception of the FDM process, extensive research has been conducted into how to improve the surface finish of FDM parts, although some degree of surface roughness is unavoidable. Many researchers have been successful in controlling and reducing surface roughness using different techniques [8]. Even in the literature, pre-processing techniques are collected for the improvement of this low surface quality. For these reasons, pre-processing and post-processing techniques must be considered [9]. On the one hand, pre-processing technologies are understood as those utilized prior to the construction of the part, that is, in the definition of slicing [10,11] or manufacturing parameters [12,13]. On the other hand, post-processing refers to any activity carried out after the manufacture of an element. These post-processing techniques could be classified into mechanical [14,15], thermal [16,17], and chemical [18,19] treatments, the latter being the fastest procedure. For example, application of an acetone vapor smoothing to *acrylonitrile butadiene styrene* (ABS) parts has been reported to reduce the surface roughness up to 90% in just ten seconds [20]. However, in most cases, these studies have not been focused on the treatment of PLAs. Therefore, their results are not of direct relevance to this research.

In addition, few studies show the impact of these treatments on other material properties. Chemical treatments can significantly alter the nature of the material and, together with it, the complete functionality of the piece [21,22]. Specifically, PLA presents an unusually wide range of properties between its amorphous state and its crystalline state. These properties can be achieved by manipulating the mixtures between the d (−) and l (+) isomers, the molecular weights, and the copolymerization [23]. Also, chemical treatments significantly affect the nature of this material, affecting in turn the functional characteristics of the parts [18,24].

In this particular work, the immersion of PLA-FDM pieces in different organic solvents during a controlled period is studied to improve the quality of the parts’ surfaces. As a result, not only is an improvement of the surface finish obtained, but a partial and relatively homogeneous crystallization of the material as well. This has not been analyzed in previous studies. With this crystallization, thermal and mechanical behavior change, thus improving resistance to temperature and to degradation.

In particular, nuclear magnetic resonance spectroscopy (NMR), X-ray diffraction (XRD), thermogravimetric analysis (TGA), and differential scanning calorimetry (DSC) analyses were obtained from the PLA manufactured pieces before and after treatment. All the observed changes could result in very different applications for the same material and process, depending on the type of treatment applied.

## 2. Materials and Methods

The material used in the development of this work was natural, or colorless, PLA. The manufacturer of this thermoplastic material, FFF World, assures that the filament is made only with virgin pellets of PLA NatureWorks (Minnetonka, MN, USA), with a proportion of less than 1% of stabilizing additives.

An FDM test bench was used for sample fabrication—prismatic specimens with a square base of 30 mm sides and 5 mm thickness. The main parameters used in the manufacture of the samples are collected in the Table 1.

Once the parts were manufactured, they were weighed and then immersed for 30 and 60 s in 4 organic solvents: Ethyl acetate (C_4_H_8_O_2_), tetrahydrofuran (C_4_H_8_O), dichloromethane (CH_2_Cl_2_), and chloroform (CHCl_3_). Three test parts were manufactured for each combination of the above-mentioned parameters. Some of these solvents have been used in other studies, and showed a favorable behavior for the improvement of the surface quality of the parts manufactured with FDM [24,25]. After the treatment they were weighed again.

The surface quality of the samples treated in each solvent for different time intervals was evaluated through the roughness profiles.

Different methods were used to characterize the material before and after post-processing. In the method explained above, the chemical composition, crystalline structure, and thermal properties of the manufactured parts were analyzed through NMR, XRD, DSC, and ATG analyses. Three similar samples (same parameters) were made for each study, as mentioned above. In all cases the averages of the values obtained are included, considering the deviation as insignificant (<5%)

NMR spectra of 1H and 13C {1H} of the samples were performed at 25 °C in deuterated chloroform (CDCl_3_) in an Agilent MR400 spectrometer from Agilent Technologies (Santa Clara, CA, USA), operating at the frequency of 500 MHz for 1H, and 125.7 MHz for 13C {1H}. X-ray diffraction diagrams of the samples were also recorded on a Bruker (Billerica, MA, USA) D8 Advance A25 diffractometer with copper anode at 40 kV and 40 mA and a nickel filter. The goniometer had a radius of 280 mm and a step size of 0.02°.

For the evaluation of the thermal stability of the polymer and the possible inclusion of residues, a thermogravimetric analysis was carried out by means of a TA-INSTRUMENTS-WATERS LLC (New Castle, DW, USA) model TGA Q50 V20.13 system.

Two thermal cycles were performed for each sample. For the first cycle, a nitrogen stream at 40 mL/min and an air stream of 60 mL/min were used as balance gas. Temperature was increased from 25 to 250 °C and then reduced to 35 °C with a heating rate of 10 °C/min. Then, a second cycle started with a nitrogen gas stream of 25 mL/min. The heating and cooling rate was 5 °C/min for an interval between 35 to 250 °C and finally back to 35 °C [26]. DSC measurements were performed using a TA-INSTRUMENTS-WATERS LLC (New Castle, DW, USA) model DSC Q20 V24.11 system.

From XRD diagrams it was possible to calculate the crystallite dimension using the Scherrer equation shown below (2). In addition, the approximate crystallization percentage was estimated by image analysis by calculating the area enclosed under the shoulder of a phase contrasted with the crystalline peak of that same phase. These areas could be compared with diagrams obtained by differential scanning calorimetry (DSC) in order to make a more accurate estimation. Free image processing software, Imagej (developed at the National Institutes of Health (Bethesda, MA, USA)), was used to calculate the areas and estimate this crystallization value.

## 3. Results and Discussion

### 3.1. Nuclear Magnetic Resonance Spectroscopy

Analyses of NMR spectra of 1H and 13C {1H} from all samples were found to be identical to each other and to those found in other studies [27,28]. It is understood, then, that the application of a bath to the solvents does not affect the composition of the material.

### 3.2. X-ray Diffraction

**Figure 1:**
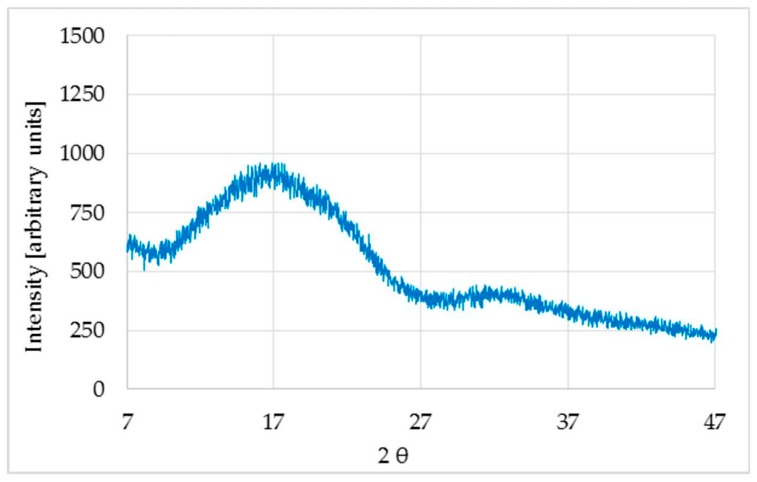
X-ray diffraction diagram of the untreated sample.

XRD of the untreated sample was analyzed and two wide bands, characteristics of an amorphous polymer, were observed (Figure 1).

The angles, 2θ, for the maximum intensity, as well as the corresponding reticular spacing, *d*, result in the application of Bragg’s law Equation (1),

(1) λ=2 d sin θ

The specific angular values are shown in Table 2.

The first spacing was found to be about twice the second spacing, meaning that they most likely correspond to the same phase of the polymer. Therefore, using Scherrer’s Equation (2) to calculate the size of the crystallite dimension along the direction perpendicular to the crystallographic plane (*hkl*), a value of about 1 nm was obtained.

(2) $$L_{hkl}\;=\;\frac{0.89\;\lambda }{\beta \cos\theta }$$

**Figure 2:**
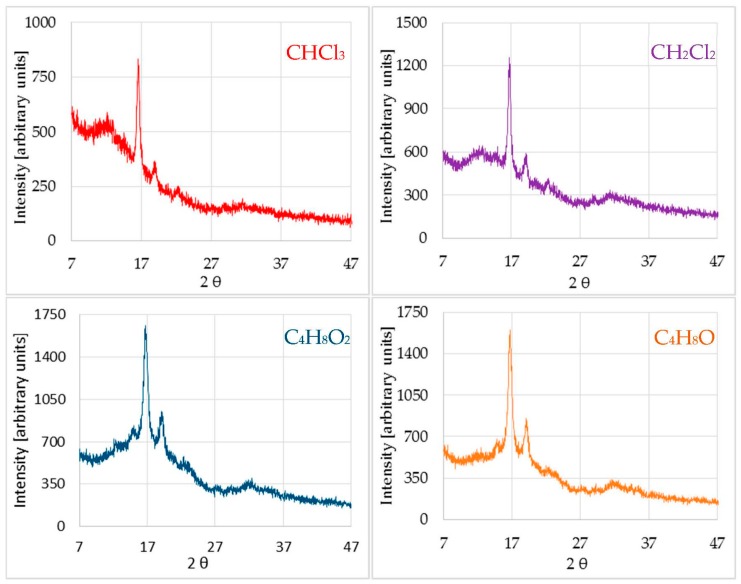
X-ray diffraction diagram of post-treated samples.

Nonetheless, XRD of the previous sample after treatment with different solvents, besides partially preserving the bands of the amorphous form, presented new peaks characteristic of a new more crystalline phase (Figure 2).

To identify the crystalline phase that appeared among the forms described in other works (α, β, δ (or α’), γ) [29], the same procedure was carried out. Results are listed in Table 2.

After treating the material with chloroform, in addition to the pre-existing amorphous phase, a new crystallized phase of the polymer was created. In principle, this could be identified as the crystalline phase referred to in the literature as α’, an α variant of which is fundamentally distinguished from it by the peak value at 2θ = 22.3°. The peak at 15°, which was not clearly observed, seemed to slightly emerge in the amorphous band (Figure 2).

The shoulder that characterizes the amorphous band was wider in ethyl acetate and in tetrahydrofuran compared to the peaks that appear in each diagram, indicating that the polymer had crystallized to a lesser extent than in the case of halogenated solvents, where the relationship between the peak and the shoulder areas was much greater. Moreover, from the data obtained and comparing to the bibliography, it appears that dichloromethane and tetrahydrofuran had provoked a partial crystallization of the material in the same phase as chloroform (α’), while ethyl acetate seemed to crystallize the material in an α-phase. However, different percentages of crystallization were obtained, with the presence of different phases and with different sizes of crystallite, resulting in very different properties, as emphasized before.

### 3.3. Thermogravimetric Analysis

The TGA results have been analyzed and the total weight variation (ΔW) calculated for each of the samples (Table 3).

At 250 °C the weight loss of the untreated sample was about 1%. Most of this loss occurred even below 100 °C. For this reason, everything seems to indicate that it corresponded to very volatile absorbed vapours, mainly water. With prolonged heating to 250 °C, the weight loss reached 2.4%. This last continuous weight loss must be attributed to either the volatility of the polymer or to slow combustion under the airflow.

The TGA of samples treated with solvents showed a significant weight loss. Up to 250 °C, the loss for the sample treated with chloroform was around 16%. This seems to indicate that the presence of very volatile and flammable solvent residues could make the polymer more volatile and/or combustible in the air stream in which the experiment was performed. However, when this weight loss was compared to the increase in weight or absorption of the polymer once treated, a lower weight loss was observed than that absorbed during the corresponding chemical treatment, with the exception of the sample treated with ethyl acetate, where the loss was similar to the gain. This could mean that the solvent was partly retained inside the sample.

### 3.4. Differential Scanning Calorimetry

**Figure 3:**
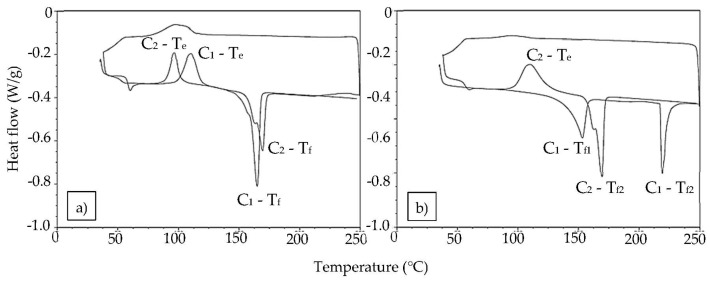
Differential scanning calorimetry analysis of the two thermal cycles (C_1_ and C_2_) for arbitrary samples: (**a**) untreated sample; (**b**) sample treated with CHCl_3_.

The graphical representation of the differential scanning calorimetry (DSC) analysis of the untreated sample is shown below (Figure 3). An exothermic glass transition and, at higher temperature, an endothermic fusion process is observed. The fact that the fusion endothermic band appears almost unfolded indicates that two phases coexisted while, in the second cycle, the proportion of the amorphous or more disordered phase decreased [30]. From this graph the numerical data of evaporation (*T*_e_), vitreous transition (*T*_g_), and fusion (*T*_f_) temperatures were collected (Table 4).

**Figure 4:**
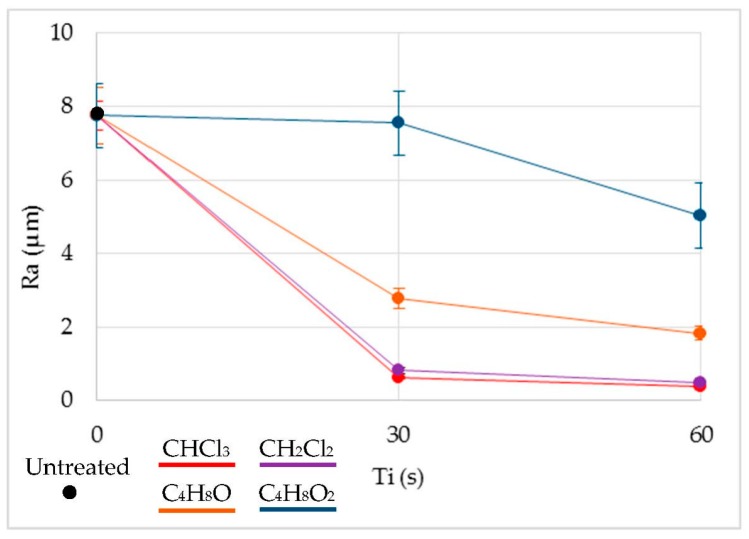
*R*_a_ as a function of immersion times (Ti) and the different solvents.

However, this behavior varied according to the treatment applied. For example, in the case of chloroform, the first heating-cooling cycle produced a very different result than in the case of the untreated sample (Figure 4). In the treated sample, the glass transition process did not occur, and instead it showed two endothermic fusion processes as an indication that an amorphous (*T*_f1_ = 153.4 °C) and a crystalline (*T*_f2_ = 219.3 °C) phase coexisted. The most significant data are shown in Table 4. It can be seen that the crystalline phase supported a much higher temperature, helping the amorphous phase to remain stable until the approximate melting temperature of the PLA was reached, so that a glass transition did not appear. This means that PLA parts coated in CHCl_3_ withstand a higher service temperature than commercial PLA, which could be interesting for many applications.

Each solvent seemed to affect the PLA differently. Treatment with CH_2_Cl_2_ caused the glass transition phase to disappear again, just as with the other halogenated solvent studied and commented on. However, in this case, the amorphous and crystalline phases melted almost simultaneously. This probably occurred because, although the amorphous phase was dominant, the heating rate applied to the samples was too fast to separate both fusion processes.

Treatment with tetrahydrofuran clearly differentiated the vitreous from the fusion transition. This may be due to the fact that the concentration of the crystallites, with respect to the amorphous part of the material, was less homogeneous, with a more separated arrangement of the phases, which enhanced its resistance to temperature changes. Finally, the fraction that crystallized in samples treated with ethyl acetate seemed to be smaller compared to the rest, showing the same behavior as the untreated amorphous polymer. Only in the second cycle did it seem to worsen its thermal properties.

In summary, treatment with chloroform and tetrahydrofuran provided greater thermal resistance to the pieces, mainly associated with the arrangement of the formed crystallites.

Nevertheless, the behavior during the second thermal cycle was very similar to that of the untreated sample, so the crystallization induced by the solvent treatment is annulled by the heating cycle. The fact that the endothermic band associated with the fusion transition appears almost unfolded in the second cycle indicates that two phases coexist, as also occurs in the untreated sample. Halogenated solvents show the highest thermal resistance in a second heating cycle by a significant amount, and this can be associated with the existence of a crystalline memory.

Finally, the melting temperature increased with the degree of crystallinity and with the arrangement of the molecular chains. However, it must be mentioned that, from the point of view of thermoplasticity, the crystallization of the phase α was not a positive fact [29].

### 3.5. Estimation of the Percentage of Crystallization

The percentage of crystallized material in the samples was obtained from the graphs of both XRD and DSC (Table 5) as has been explained in the methodology. Partial crystallization is an important indicator and can be measured more accurately by calculating the enthalpies (melt peak and cold crystallization peak) [26]. However, the approximation provided by this method is considered sufficient for the purposes of this work, because the influence of these post-treatments on the material has not been analyzed in previous studies.

Halogenated solvents crystallized the material to a greater extent than non-halogenated ones. In all cases, however, the size of the crystalline domains did not significantly differ, being of an order of magnitude greater than those of the amorphous polymer, as discussed above. Control of this crystal formation can be very interesting to regulate material properties.

### 3.6. Roughness

**Figure 5:**
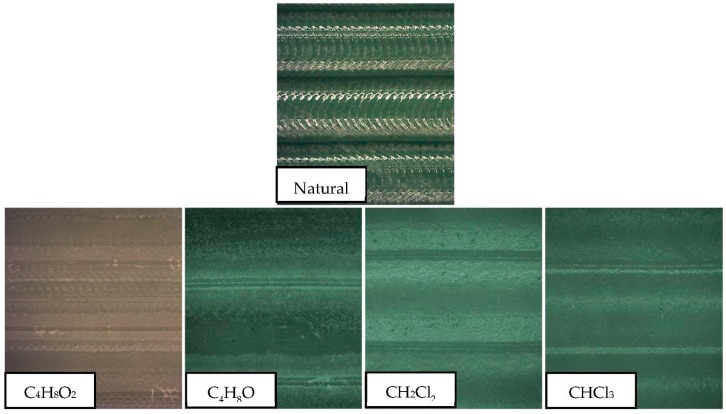
Surface smoothing after immersion in solvents.

The term arithmetic means roughness (*R*_a_) was analyzed as the main parameter for the evaluation of the surface quality. Figure 4 shows the results of *R*_a_ prior to treatment and once immersed in each solvent. The immersion time did not seem to be a very significant variable in comparison to the type of solvent used. Thus, improvements of *R*_a_ of up to 97% were obtained in the case of chloroform, 94% in the case of dichloromethane, almost 80% in the case of tetrahydrofuran, and 35% in the case of ethyl acetate. Effects on the smoothing of the surface can be seen in Figure 5.

The irregularities at the surface trapped the volatile solvent, making the surface material viscous or semi-fluid by absorption of the volatile solvent. For this reason, under the effects of surface tension and gravity, the surface irregularities were diminished until dynamic equilibrium was achieved, resulting in a smoother surface.

A similar behavior can be established in the treatment governed by halogenated solvents. In both cases, *R*_a_ decreased drastically, until it reached surface qualities unattainable from the process and from other polymeric manufacturing processes. The chlorine contained in both compounds tried to dissolve the polymeric material, invading its structure. However, with such a short exposure time, the material did not dissolve but was redistributed, resulting in a smoother and more homogeneous surface. Therefore, the roughness was reduced. Non-halogenated solvents also reduce the *R*_a_, although results do not reach those obtained with the halogenated liquids. In these cases, time does seem to be a more significant variable in the process in the range studied, although ethyl acetate shows no substantial improvement in comparison with the rest of the solvents used.

Also, *R*_a_ converged to a minimum after a certain immersion time, after which no improvement in the surface smoothing was found. If the material was exposed for longer times, the softening of the material would be so general that the morphology of the surface or of the whole piece would be affected.

## 4. Conclusions

Immersion in solvents changes the characteristics of manufactured PLA pieces. The treatments do not significantly alter the chemical nature of the PLA polymer according to NMR analysis, although they do modify the microstructure of the material and their thermal properties. These changes could significantly affect the mechanical properties of the parts. In addition, variations in mass associated with the absorption of these solvents and water, probably coming from the environment, are verified, which means the absorption of solvent and/or humidity occludes inside the porosities, since it would volatilize on the surface.

It also appears that halogenated solvents crystallize the material to a greater extent than non-halogenated solvents. However, the size of the crystalline domains does not differ greatly in all cases.

This partial crystallization does not appear to be directly related to the increase in temperature resistance for some cases. Halogenated solvents cause the sample to crystallize at a relatively high percentage, and the arrangement of these chains seems to prevent a glass transition phase from appearing. In this way, the temperature resistance of the parts rises almost to the original melting temperature of the material. In addition, for chloroform, two fusion transitions are clearly differentiated, each of them associated with the amorphous and crystalline parts respectively. However, in the case of dichloromethane, these two phases seem to be grouped together, causing a simultaneous fusion of all the material.

On the other hand, non-halogenated solvents, while providing partial crystallization in the material, do not seem to increase the resistance to the temperature of the samples. In particular, ethyl acetate even worsens this property. In this case, the phase that appears in the polymer with the application of these new treatments is not the same as for the previous cases.

In summary, an improvement in surface quality of the FDM process is achieved by immersion in organic solvents. This procedure, though, changes certain material properties, making it possible to obtain a wider range of them and delay the degradation of the parts. For this reason, a balance must be made between the characteristics intended to be improved.

Pieces made with PLA and immersed in chloroform present better surface quality and thermal characteristics and greater crystallization compared to those studied in this work. For this reason, polluting polymers could be substituted by PLA in order to make the manufacture of replacing parts more eco-friendly.

Future work could analyze how this procedure affects the macro geometrical characteristics of the manufactured pieces. Specifically, internal modifications in FDM printed parts, including interfacial bonding, could be of interest.

## Figures and Tables

**Table 1:** Values of the most characteristics fixed parameters.

Sample	Parameter	Value
	Nozzle diameter	0.4 mm
	Layer thickness	0.2 mm
	Speed	20 mm/s
	Overlap	55%
	Nozzle temperature	220 °C
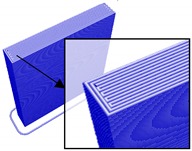	Bed temperature	65 °C
	Retraction	1.7 mm
	Retraction speed	35 mm/s
	Infill	100%
	Infill pattern	Rectilinear
	Infill angle	0°

**Table 2:** Angular values of diffraction maxima and reticular spacing in the sample.

Sample	Bands	2θ [°]	d (nm)	L_hkl_ (Å)
Natural	Band 1	17.24	0.51	9.74
	Band 2	33.15	0.27	13.64
	Band 1	16.61	0.53	147.28
CHCl_3_	Band 2	18.90	0.47	85.38
	Band 3	22.31	0.39	104.29
	Band 1	15.21	0.58	188.54
CH_2_Cl_2_	Band 2	18.48	0.48	142.12
	Band 3	22.40	0.39	250.43
C_4_H_8_0	Band 1	15.21	0.58	143.84
	Band 2	19.11	0.46	136.96
C_4_H_8_O_2_	Band 1	16.73	0.53	118.62
	Band 2	19.05	0.46	104.87

**Table 3:** Weight gain with post-treatment application and thermogravimetric analysis.

	Test	Natural	C_4_H_8_O_2_	C_4_H_8_O	CH_2_Cl_2_	CHCl_3_
ΔW [%]	Post-treatment	-	3.7	7.1	10.4	16.0
	TGA	−2.4	−3.8	−6.8	−7.8	−15.1

**Table 4:** Numerical data from the differential scanning calorimetry analysis of the samples.

Sample	Cycle	*T*_e_ [°C]	*T*_g_ [°C]	*T*_f1_ [°C]	*T*_g2_ [°C]	*T*_f2_ [°C]
Natural	1	59.7	110.7	169.5	-	-
	2	-	96.9	165.0	-	-
CHCl_3_	1	-	-	153.4	-	219.3
	2	-	110.0	169.2	-	-
CH_2_Cl_2_	1	-	-	166.5	-	-
	2	-	105.8	169.3	-	-
C_4_H_8_O	1	59.5	102.4	161.5	197.1	238.6
	2	-	96.9	165.7	-	-
C_4_H_8_O_2_	1	59.2	102.2	165.5	-	-
	2	-	86.1	159.9	-	-

**Table 5:** Approximate percentage of crystallized sample.

	Natural	C_4_H_8_O_2_	C_4_H_8_O	CH_2_Cl_2_	CH_3_Cl_3_
Crystallization [%]	≈0	12.15	24.60	38.5	41.70
